# Highlight: On the Origin of the Sexes

**DOI:** 10.1093/gbe/evt164

**Published:** 2013-11-10

**Authors:** Danielle Venton



In an 1860 letter to his friend Reverend J. S. Henslow, Charles Darwin speculated that the existence of two sexes, male and female, was the greatest mystery in the world. Especially since the discovery of asexual reproduction by parthenogenesis, he wrote, “the origination of the sexes seems beyond all speculation.”

New work by a research team lead by John Allen, professor of biochemistry at Queen Mary University of London, proposes an answer. “I think we may have a principle here,” says Allen, “an explanation of why we have two sexes, male and female.”

Examining the metabolic rates of mitochondria in male and female germlines, the researchers present evidence that females do indeed hold “template mitochondria” by carrying non-respiring mitochondria. Unlike females, male germlines have active mitochondria—sperm, after all, must swim. These findings hold true even in animal species separated by hundreds of millions of years of evolution: jellyfish, fruit flies, and zebra fish.

“This could give us a new idea on why you have different sexes,” says Carl Bauer, a biochemistry professor at Indiana University, Bloomington, not involved in the research, “and how far the body goes to protect the mitochondria for the next generation.”

The current work published in *Genome Biology and Evolution* (de Paula et al. 2013) by Allen and his team was designed explicitly to test a theory of Allen’s proposed in the mid-90s. Allen’s (1996) background is primarily in biochemical world of plants, chloroplasts especially. But about 20 years ago, he turned his mind to the animal kingdom. “It occurred to me,” he says, “animals pay a huge cost to retain genes in mitochondria.”

In the cell, it is the mitochondrion’s job to generate most of the cell’s energy needs, in the form of adenosine triphosphate (ATP). The process of respiring oxygen (known as oxidative phosphorylation) creates oxygen free radicals which, in their hunger for electrons, have the tendency to rip DNA to shreds and cause mutations. Yet, some DNA is stored here.

“The mitochondrion is about the worst imaginable place in the cell to keep genetic information,” Allen says. “It’s like keeping all your precious documents next to the incinerator.”

One school of thought, known as the mitochondrial theory of aging, holds that the organisms grow old because of the mutations that accumulate in their mitochondrial DNA. Diseases such as Pearson syndrome, Leigh syndrome, some cardiomyopathies, Alzheimer’s disease, and Parkinson disease can be caused by faulty mitochondrial DNA rearrangements. The mutation probably happens in the female germ line or in embryonic development—but there is a mystery: few mutations are transmitted to each successive generation. Offspring inherit their mother’s mitochondria but do not inherit her accumulated mutations. It is as if the clock is reset.

Allen’s theory published in the *Journal of Theoretical Biology* in 1996 proposed that the female germline holds a special, quiescent line of “template” mitochondria. These organelles, Allen proposed, are transcriptionally and energetically repressed, accumulating far fewer mutations, and provide an accurate source of information for mitochondria in the next generation.

“[Allen] goes for broad questions,” says Wilson de Paula, a doctoral candidate in Allen’s lab. “He’s one of the very few scientists who proposes theories that are easy to check in the lab.”

The work of de Paula, Allen and others demonstrate that in oocytes the mitochondria are transcriptionally inactive, have decreased membrane potential, and produce fewer reactive oxygen species. Although the findings have struck many in the community as novel, de Paula says the work echoes findings from the 1970s.

“Those papers didn’t get much attention,” says de Paula, adding that the medical field has erroneously pushed the idea that only an active, ATP-producing oocytes are healthy.

Though that is wrong, he says, it’s true the oocyte must break its dormancy at some point, become active, and differentiate into the forming embryo. Understanding how this dormancy is broken could, he hopes, one day shed light on why some pregnancies fail. Before that, he cautions, there is much other work to be done.

“It’s very interesting on a number of levels,” says Neil Blackstone, evolutionary biology professor at Northern Illinois University not involved in the work. “Evolutionary biologists rarely focus on mitochondria or think about metabolism—metabolism is key here.”

Blackstone is eager to see the evolutionary generality of the team’s findings. “They’ve done wonderful work on three species and this finding may be general,” he says, “but other mechanisms could potentially explain this too.”

Blackstone raises the possibility that stem cells, which also seem capable of escaping the trappings of aging, might also be metabolically quiescent. If so, he wonders, “it could lead to a different way of looking at stem cells.”

Carl Bauer of Indiana University is curious to know whether this quiet mitochondrial pattern holds true in mammalian oocytes as well.

“Over time I think the evidence is starting to weigh in that [the mitochondrial theory of aging] is a very reasonable hypothesis,” he says. In rare cases where children inherit mitochondria from their fathers, the offspring seem to age prematurely. Dolly the sheep was cloned from an adult cell (inheriting old mitochondria) and likewise prematurely aged and died.

“One of the things this work has not yet addressed,” says Bauer, “is the means to regulate this. How do you selectively turn off gene expression? It’ll be fascinating to see how this plays out.”

The work of Allen, de Paula and others may, Bauer believes, “lead to a complete change in thinking—giving us a new idea on why you have different sexes and how far life goes to protect mitochondria for the next generation.”
